# Ebola GP-Specific Monoclonal Antibodies Protect Mice and Guinea Pigs from Lethal Ebola Virus Infection

**DOI:** 10.1371/journal.pntd.0001575

**Published:** 2012-03-20

**Authors:** Xiangguo Qiu, Lisa Fernando, P. Leno Melito, Jonathan Audet, Heinz Feldmann, Gary Kobinger, Judie B. Alimonti, Steven M. Jones

**Affiliations:** 1 Special Pathogens Program, National Microbiology Laboratory, Public Health Agency of Canada, Winnipeg, Manitoba, Canada; 2 Department of Medical Microbiology, University of Manitoba, Winnipeg, Manitoba, Canada; 3 Department of Immunology, University of Manitoba, Winnipeg, Manitoba, Canada; University of Texas Medical Branch, United States of America

## Abstract

*Ebola virus* (EBOV) causes acute hemorrhagic fever in humans and non-human primates with mortality rates up to 90%. So far there are no effective treatments available. This study evaluates the protective efficacy of 8 monoclonal antibodies (MAbs) against Ebola glycoprotein in mice and guinea pigs. Immunocompetent mice or guinea pigs were given MAbs i.p. in various doses individually or as pools of 3–4 MAbs to test their protection against a lethal challenge with mouse- or guinea pig-adapted EBOV. Each of the 8 MAbs (100 µg) protected mice from a lethal EBOV challenge when administered 1 day before or after challenge. Seven MAbs were effective 2 days post-infection (dpi), with 1 MAb demonstrating partial protection 3 dpi. In the guinea pigs each MAb showed partial protection at 1 dpi, however the mean time to death was significantly prolonged compared to the control group. Moreover, treatment with pools of 3–4 MAbs completely protected the majority of animals, while administration at 2–3 dpi achieved 50–100% protection. This data suggests that the MAbs generated are capable of protecting both animal species against lethal *Ebola virus* challenge. These results indicate that MAbs particularly when used as an oligoclonal set are a potential therapeutic for post-exposure treatment of EBOV infection.

## Author Summary

*Ebola virus* (EBOV) causes acute hemorrhagic fever in humans and non-human primates with mortality rates up to 90%. So far there are no effective treatments available. This study evaluates the protective efficacy of 8 monoclonal antibodies (MAbs) against the *Ebola virus* surface glycoprotein, in mice and guinea pigs. Various combinations and doses of the neutralizing and non-neutralizing MAbs were tested, and a post-exposure treatment protocol was determined. There was 100% survival when guinea pigs received a mix of 3 neutralizing MAbs two days after a challenge with 1,000 LD_50_ of guinea pig-adapted EBOV. This data suggests that the MAbs generated are effective as a post-exposure therapeutic for a lethal Ebola virus infection. Development of a post-exposure therapeutic for an *Ebola virus* infection is vital due to the high lethality of the disease, the relative speed in which it kills, and the fact that no vaccine has been approved for human use. Additionally, is it unlikely that preventative vaccines will be employed, because Ebola virus infections occur primarily in Africa, and to date have only killed approximately 2,300 people making it financially unfeasible for a mass vaccination. Therefore, having an effective therapy in the event of an outbreak would be extremely beneficial.

## Introduction

*Ebola virus* (EBOV) is a filovirus causing severe viral haemorrhagic fever in humans and non-human primates (NHPs) [1]. There are five species of EBOV: *Zaire ebolavirus* (ZEBOV), *Sudan ebolavirus* (SEBOV), *Cote d'Ivoire ebolavirus* (CIEBOV), *Reston ebolavirus* (REBOV), and *Bundibugyo ebolavirus* (BEBOV) [2]. ZEBOV has the highest virulence with a case fatality rate of 60–90% [1], [3]. Although several attempts have been made to treat EBOV infections [4]–[8], there are currently no commercially approved vaccines or effective therapies, therefore new treatments are needed. Several studies have been conducted to determine the immune correlates of protection in EBOV infections either by following natural infections, or in *in vivo* animal models [9]–[15]. Both T and B cell immunity was analysed and it was believed that a strong early humoral immune response may have been a factor in survival [11], [16], [17]. Additionally, in fatally infected patients EBOV-specific IgG was absent, and IgM levels were low in comparison to the survivors [16]. The passive transfer of immune sera or whole blood was tested but its effectiveness is still controversial as it has not consistently provided protection [10]. However, in mice experiments EBOV-specific sera was sufficient for improving survival [10], [18], [19].

The key target for developing effective neutralizing antibodies (NAb) is suspected to be the surface glycoprotein (GP) [20]. EBOV GP is the only protein on the surface of the virus and is responsible for receptor binding, viral entry, and cellular tropism [20]–[24]. GP-specific NAb generated in several species were protective in some animal models, however, the NAb titres are low in natural infections and their effectiveness in humans remains to be confirmed [10], [25]–[27]. Antibodies blocking viral entry, by binding the receptor or preventing viral fusion would be ideal candidates for improving survival. Additionally, the primary pathology of EBOV haemorrhagic fever is vascular injury and coagulation abnormalities, and GP has been shown to cause cytotoxicity and vascular permeability [28], [29]. In fact GP-induced cytotoxicity has been correlated with mortality rates in the different EBOV viral species [28], [30]. Taken together this suggests that prophylactic and post-exposure treatment strategies involving antibodies specific for the EBOV GP would be an effective intervention for an Ebola infection.

Monoclonal antibodies (MAbs) against ZEBOV GP have been created previously and tested in several animal models as a post-exposure therapeutic [26], [27], [31]–[34]. However, the ability of each of the MAbs to improve survival in a lethally infected animal varied considerably. Some MAbs were able to protect mice completely yet guinea pigs partially [32], [33]. One neutralizing MAb KZ52 was 100% efficacious in guinea pigs, but did not protect NHPs [26], [35]. Overall, there are a variety of mechanisms employed by MAbs to improve survival, and the ability of the MAb to neutralize the virus is not essential. The MAbs tested so far are not 100% efficacious in all animal models therefore further research is needed for more effective antibodies. The goal of this study was to test a panel of MAbs specific for the ZEBOV GP for their efficacy in protecting mice and guinea pigs from a lethal ZEBOV infection. Previously, 8 ZEBOV GP-specific MAbs had been generated using the VSVΔG/ZEBOVGP vaccine as the immunogen [36]. A preliminary study characterizing the MAbs found they all improved survival in mice infected with a high dose of mouse adapted-ZEBOV (MA-ZEBOV) [36]. As the MAbs were effective in the mouse model it is possible that these MAbs could be used as a post-exposure therapeutic for a ZEBOV infection. In this study optimization of a post-exposure protocol is undertaken in both the mouse and guinea pig model in order to determine the various treatment parameters, including the dose, treatment time, and MAb combination, that are required to provide complete protection.

## Materials and Methods

### Ethics Statement

All infectious animal work was performed in the biosafety level 4 biocontainment laboratory at the Public Health Agency of Canada, and approved by the Canadian Science Centre for Human and Animal Health Animal Care Committee following the guidelines of the Canadian Council on Animal Care. Animals were acclimatized for 10 days prior to the start of the experiment, and fed and monitored daily pre- and post-infection.

### Viruses

The recombinant virus VSVΔG/ZEBOVGP containing the *Zaire ebolavirus*, strain Mayinga, glycoprotein (GP) in place of the VSV glycoprotein (G) has been described previously [37]. The mouse-adapted (MA-ZEBOV) and guinea pig-adapted (GA-ZEBOV) ZEBOV strain Mayinga viruses were described previously [38], [39].

### Mice Immunization and MAb Production and Purification

The creation of 8 MAbs (1H3, 2G4, 4G7, 5D2, 5E6, 7C9, 7G4, 10C8) has been described previously [36]. Briefly, 6–8 week old Balb/C mice were immunized with 10^7^ pfu VSVΔG/ZEBOVGP intraperitoneally (ip), at 0, 4, and 8 weeks. A final boost with *Zaire ebolavirus* like particles (eVLPs) was performed before harvesting spleen cells and fusing with SP2/0 myeloma cells according to Kohler and Milstein [40]. The generation of the ZEBOV GP/VP40 eVLPs have been described previously [41].

The hybridomas were grown in Hybridoma SFM (Invitrogen), 1 mM L-Glutamine, 1× Antibiotic-Antimycotic (Invitrogen), in roller bottles at 37°C, 5% CO_2_. Supernatant was cleared by centrifugation and concentrated ten times using an Amicon Stirred Cell system with a 30 kDA MWCO filter (Millipore). The antibodies were purified on a HiTrap Protein G HP column(GE Healthcare) using Protein A Binding Buffer and IgG Elution Buffer (Thermo Scientific) according to manufacturers' instructions. Positive fractions were pooled, concentrated, then buffer exchanged into PBS using a 10 kDa MWCO Centriprep unit (Millipore). Antibody purity, assessed by gel electrophoresis and coomassie blue staining was >98%.

### Animal Experiments

The 5–6 week old female Balb/C mice from Charles River, (Quebec, Canada), were injected ip with the indicated amount of ZEBOV GP-specific MAbs in 100 µl PBS at the times indicated either before or after i.p. infection with 1,000 LD_50_ of MA-ZEBOV. Female guinea pigs (Hartley strain), approximately 250 g, from Charles River, were challenged with 1,000 LD_50_ of GA-EBOV i.p.. At the indicated times post-infection the guinea pigs were treated i.p. with 1 ml of the MAb diluted in PBS. Naive control animals received PBS only. Clinical signs of infection and body weight were monitored for two weeks after challenge and survivors were followed three times longer than the death of the last control animal.

### VSVΔG/ZEBOVGP Plaque Reduction Neutralization Assay (PRNT_50_)

ZEBOVGP-specific MAbs were serially diluted from 1/100–1/12,800 in DMEM. Starting concentrations were 3.75, 3.46, 4.34 mg/ml for 1H3, 2G4, and 4G7, respectively. The MAbs were added to an equal volume of 10^4^ pfu/ml VSVΔG/ZEBOVGP, diluted with DMEM, in order to provide 200 pfu/well. The virus-antibody combination was incubated at 37°C for 1 hour before adding 150 ul/well to a confluent 12 well tissue culture plate seeded with Vero E6 cells. After a 1 hour incubation, 1 ml of MEM 2% FBS, 1% low melting point agarose was added per well. Plates were incubated at 37°C 5% CO_2_ for 48 hours before adding 1 ml of 0.2% w/v crystal violet, 3.7% Formaldehyde, 2% Ethanol to each well for visualization of the plaques. The assay was performed in triplicate, and a positive control (virus with no antibody) and a negative control (no virus) incorporated. The percent reduction was calculated by averaging the count of the triplicate wells and comparing the number of plaques in the test sample against the number of plaques in the positive control (1−(Test Sample plaques/Positive control plaques))×100 = % reduction.

### Statistics

The log rank statistical test was performed for the Survival curve using the GraphPad Prism 4 software program. The survival curve for the MAb treated animals were compared to the survival curve for the PBS control group.

## Results

### Characterization of MAbs

**Figure 1:**
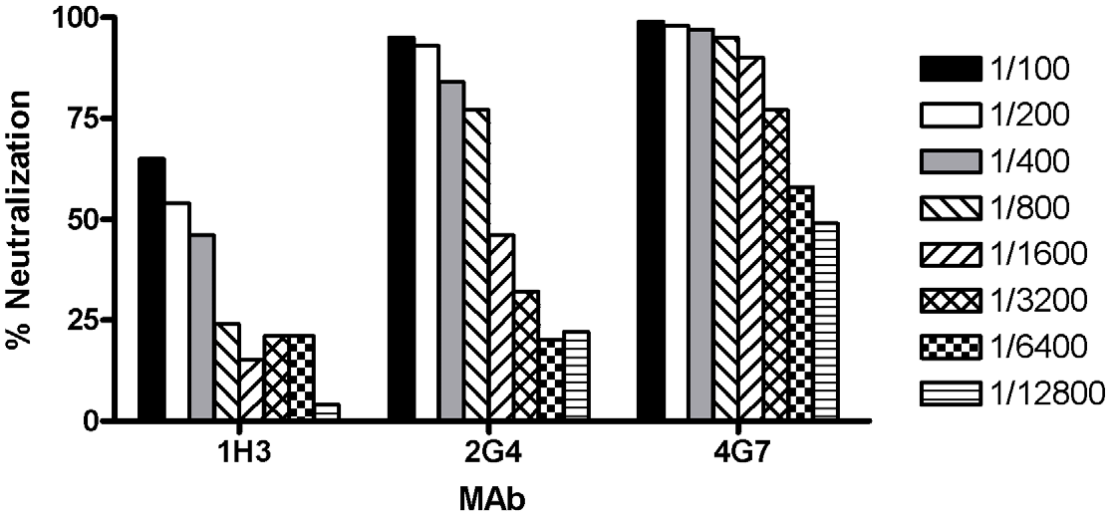
*PRNT_50_ assay for MAbs*. ZEBOVGP-specific MAbs were serially diluted, and added to an equal volume of VSVΔG/ZEBOVGP for 1 hour at 37°C before adding to a confluent 12 well tissue culture plate seeded with Vero E6 cells. After 2 days the plaques were stained and counted. The percent reduction was calculated by determining the percent reduction in plaques at each dilution in comparison to the positive control (virus with no antibody). The assay was performed in triplicate.

Previously, 8 MAbs specific for the glycoprotein (GP) of ZEBOV had been generated [36]. An initial characterization demonstrated they bound to a variety of GP segments, and that all 8 MAbs were able to pull down ZEBOV GP1,2 in an immunoprecipitation assay. In the current study, we further characterized the MAbs using a plaque reduction neutralization assay (PRNT_50_). The PRNT_50_ demonstrated that MAbs 1H3, 2G4, and 4G7 were neutralizing with a PRNT_50_ at a 1/200, 1/800, and 1/6,400 dilution, respectively (Figure 1). All of the other MAbs were non-neutralizing with 5D2 showing the highest degree of neutralization at 38% (data not shown).

### Protective Efficacy of MAbs in Mice

Preliminary experiments in mice suggested these MAbs would be effective as a therapeutic for a MA-ZEBOV infection [36]. Therefore, a variety of parameters were assessed in order to establish the most effective treatment protocol. An *in vivo* mouse model was utilized to determine the protective efficacy of the individual MAbs (Table 1). Each MAb was injected either 1 day before (−1) or after (+1) a MA-ZEBOV infection (1,000 LD50) in Balb/C mice. All control mice receiving PBS only had a mean time to death of 6.6 and 5.0 days for the −1 and +1 day treatment, respectively. In contrast, mice treated with MAbs (100 µg) demonstrated either partial or complete protection. For the −1 day protocol, the MAbs 5D2, 5E6, 7C9 were most effective with a 73–87% survival rate, in comparison to the other 5 MAbs (1H3, 2G4, 4G7, 7G4, 10C8) where survival rates ranged from 0–7%. Alternatively, every MAb performed better when given at 1 day post-infection (dpi), with survival rates ranging from 40–100%. Overall, the level of protection against lethality varied with each MAb, and it appears that, in general, the MAbs are more effective when given 1 day after a lethal MA-ZEBOV infection.

**Table 1:** Protective efficacy of MAbs in mice.

MAb	Treatment time^a^	Mean time to death^b^	No of survivors/total	Survival (%)
**1H3**	−1	6.60±0.61 (n = 10)	0/15	0
	+1	8.10±0.74 ( n = 9)	6/15	40
**2G4**	−1	7.86±0.74 ( n = 14)	1/15	7
	+1	8.00 ( n = 6)	9/15	60
**4G7**	−1	7.08±0.74 ( n = 14)	1/15	7
	+1	8.25±0.43 ( n = 4)	11/15	73
**5D2**	−1	8.00±1.00 ( n = 2)	13/15	87
	+1	N/A^c^	15/15	100
**5E6**	−1	8.25±0.43 ( n = 4)	11/15	73
	+1	7.00 ( n = 1)	14/15	93
**7C9**	−1	7.75±0.43 ( n = 4)	11/15	73
	+1	8.00±0.82 ( n = 3)	12/15	80
**7G4**	−1	8.07±0.59 ( n = 14)	1/15	7
	+1	N/A^c^	15/15	100
**10C8**	−1	7.64±1.17 ( n = 14)	1/15	7
	+1	8.50±0.50 ( n = 2)	13/15	87
**PBS**	−1	6.60±0.80 ( n = 5)	0/5	0
	+1	5.00±0.60 ( n = 10)	0/10	0

a Mice were treated i.p. with 100 µg of MAb at the indicated days before or after challenge with 1000 LD_50_ of the mouse-adapted Ebola virus.

b Data for animals that died (number of animals in calculation).

c N/A: not applicable.

### Dose-Dependent Protective Efficacy of the MAbs in Mice

Since the MAbs were most effective when given 1 day after the lethal MA-ZEBOV infection, this treatment protocol was used to determine the most effective dose for protection (Table 2). A dose response was observed, and some MAbs were more potent than others for a given dose. The lowest doses providing complete protection from lethality for 5D2, 5E6, 7C9, and 7G4 were 12.5, 25, 50, and 100 µg, respectively. MAbs 4G7 and 10C8 demonstrated an 83% survival rate at the highest dose of 100 µg. MAbs 1H3 and 2G4 were not included as they were not very effective at the highest dose in the first experiment (Table 1). In the partially protected groups of mice, the mean time to death ranged from 6.40 to 8.20 days in comparison to the control mice (5.80 days). Overall, the various MAbs varied in their potency in providing protection against a lethal MA-ZEBOV infection in mice.

**Table 2:** Dose-dependent protective efficacy of MAbs in mice.

MAb^a^	Dose (µg/treatment)	Mean time to death^b^ (days)	No. of survivors/total	Survival (%)
**4G7**	100	7.00 (n = 1)	5/6	83
	50	7.00 (n = 1)	5/6	83
	25	6.00 (n = 3)	3/6	50
	12.5	6.80 (n = 5)	1/6	17
	6.25	8.20 (n = 5)	1/6	17
**5D2**	100	N/A^c^	6/6	100
	50	N/A^c^	6/6	100
	25	N/A^c^	6/6	100
	12.5	N/A^c^	6/6	100
	6.25	7.50 (n = 2)	4/6	67
**5E6**	100	N/A^c^	6/6	100
	50	N/A^c^	6/6	100
	25	N/A^c^	6/6	100
	12.5	6.50 (n = 2)	4/6	67
	6.25	6.67 (n = 3)	3/6	50
**7C9**	100	N/A^c^	6/6	100
	50	N/A^c^	6/6	100
	25	7.00 (n = 1)	5/6	83
	12.5	7.00 (n = 1)	5/6	83
	6.25	6.50 (n = 4)	2/6	33
**7G4**	100	N/A^c^	6/6	100
	50	7.50 (n = 1)	4/6	67
	25	7.00 (n = 1)	5/6	83
	12.5	7.60 (n = 5)	1/6	17
	6.25	6.60 (n = 5)	1/6	17
**10C8**	100	7.00 (n = 1)	5/6	83
	50	7.00 (n = 1)	5/6	83
	25	7.50 (n = 4)	2/6	33
	12.5	7.00 (n = 5)	1/6	17
	6.25	6.40 (n = 5)	1/6	17
**PBS**		5.80 (n = 5)	0/5	0%

a Mice were treated i.p. with various doses of the MAb 1 dpi with 1000 LD_50_ of the MA-ZEBOV. The survival of the mice was followed.

b Data for animals that died (number of animals in parentheses).

c N/A: not applicable.

### Time-Dependent Protective Efficacy of MAbs in Mice

Using the most effective MAb dose of 100 µg, the treatment time was extended in both directions in order to determine the optimal time for treatment, and to see how late treatment can be given before the survival rate declines (Table 3). A single dose of 100 µg for each MAb was injected either 1 or 4 days before a lethal MA-ZEBOV infection, or at 1, 2, or 3 dpi, and survival followed. Pre-treatment of the mice 4 days before infection with 1H3, 2G4, or 7G4 did not result in survival, whereas the other MAbs provided 30–90% protection. In the majority of cases, treatment 1 day before infection resulted in lower survival rates than 4 days before infection. Of the 8 MAbs, the most effective MAbs for pre-treatment were 5D2, 5E6, and 7C9. They had the highest survival rates (73–90%) and worked almost equally well on both days −4 and +1.

**Table 3:** Time-dependent protective efficacy of MAbs in mice.

MAb	Treatment time^a^	Mean time to death^b^	No of survivors/total	Survival (%)
**1H3**	−4	6.70±0.61 ( n = 10 )	0/10	0
	−1	6.60±0.61 ( n = 10 )	0/15	0
	+1	8.10±0.74 ( n = 9 )	6/15	40
	+2	6.60±0.80 ( n = 5 )	5/10	50
	+3	6.40±0.97 ( n = 10 )	0/10	0
**2G4**	−4	7.40±0.63 ( n = 10 )	0/10	0
	−1	7.86±0.74 ( n = 14 )	1/15	7
	+1	8.00±0.00 ( n = 6 )	9/15	60
	+2	7.30±0.47 ( n = 3 )	7/10	70
	+3	5.70±1.13 ( n = 10 )	0/10	0
**4G7**	−4	7.42±0.46 ( n = 7 )	3/10	30
	−1	7.08±0.74 ( n = 14 )	1/15	7
	+1	8.25±0.43 ( n = 4 )	11/15	73
	+2	N/A^c^	10/10	100
	+3	5.67±1.34 ( n = 9 )	1/10	10
**5D2**	−4	7.00 ( n = 1 )	9/10	90
	−1	8 .00±1.00 ( n = 2 )	13/15	87
	+1	N/A^c^	15/15	100
	+2	7.00±0.00 ( n = 4 )	6/10	60
	+3	6.30±1.05 ( n = 10 )	0/10	0
**5E6**	−4	7.00±0.00 ( n = 2 )	8/10	80
	−1	8.25±0.43 ( n = 4 )	11/15	73
	+1	7.00 ( n = 1 )	14/15	93
	+2	6.00 ( n = 1 )	9/10	90
	+3	5.8±1.03 ( n = 10 )	0/10	0
**7C9**	−4	7.00 ( n = 1 )	9/10	90
	−1	7.75±0.43 ( n = 4 )	11/15	73
	+1	8±0.82 ( n = 3 )	12/15	80
	+2	7.00 ( n = 1 )	9/10	90
	+3	6.1±0.67 ( n = 10 )	0/10	0
**7G4**	−4	8.2±0.71 ( n = 10 )	0/10	0
	−1	8.07±0.59 ( n = 14 )	1/15	7
	+1	N/A^c^	15/15	100
	+2	7.10±0.57 ( n = 9 )	1/10	10
	+3	6.70±0.44 ( n = 10 )	0/10	0
**10C8**	−4	7.83±0.64 ( n = 6 )	4/10	40
	−1	7.64±1.17 ( n = 14 )	1/15	7
	+1	8.50±0.50 ( n = 2 )	13/15	87
	+2	6.83±0.37 ( n = 6 )	4/10	40
	+3	6.30±1.13 ( n = 10 )	0/10	0
**PBS**	−4	5.40±1.43 ( n = 10 )	0/10	0
	−1	6.60±0.80 ( n = 5 )	0/5	0
	+3	5.00±0.60 ( n = 10 )	0/10	0

a Mice were treated i.p. with 100 µg of each MAb at indicated days before or after infection with 1000 LD_50_ MA-ZEBOV. The survival of the mice was followed.

b Data for animals that died (number of animals in calculation).

c N/A: not applicable.

Extending the start of treatment after infection also had noticeable effects upon survival. Treating the mice on 1 or 2 dpi was the most effective treatment time, with some MAbs (5D2; 100%, 5E6; 93%, 7G4; 100%, 10C8; 87%) being more protective when given at 1 dpi, and the others (1H3; 50%, 2G4; 70%, 4G7; 100%, 7C9; 90%) when given at 2 dpi. Delaying the treatment to 3 dpi resulted in no survival for all MAbs except for 4G7 (10% survival rate). In general post-exposure treatment worked better than prophylactic treatment for the majority of the MAbs, except 5D2, 5E6, 7C9 which were highly effective at improving survival in mice both before and after the infection.

### MAb Treatment Protects Guinea Pigs from a Lethal GA-ZEBOV Infection

All MAbs were once again tested individually in the guinea pig model. The MAbs were given i.p. at 1 dpi with 1,000 LD_50_ of GA-ZEBOV, and survival followed (Table 4). The PBS controls all died with a mean time to death of 7.7 days. In those groups receiving treatment, with the exception of 2G4 and 4G7, none of the guinea pigs survived, but the mean time to death was significantly extended (range of 9.4–11.7 days, p<0.050). For 2G4 or 4G7, the survival rate was 60%, demonstrating that the MAbs can provide levels of protective efficacy individually in the more stringent guinea pig model.

**Table 4:** Prolonged survival seen in MAb-treated guinea pigs.

MAb^a^	Mean time to death^b^ (days)	No of survivors/total	Survival (%)	Student's T-test
**1H3**	11.70±2.18 ( n = 5 )	0/5	0	p = 0.018
**2G4**	11.50±1.50 ( n = 2 )	3/5	60	N/A^c^
**4G7**	10.50±1.50 ( n = 2 )	3/5	60	N/A^c^
**5D2**	9.40±1.02 ( n = 5 )	0/5	0	p = 0.024
**5E6**	10.80±1.47 ( n = 5 )	0/5	0	p = 0.009
**7C9**	9.60±0.80 ( n = 5 )	0/5	0	p = 0.006
**7G4**	9.60±0.80 ( n = 5 )	0/5	0	p = 0.006
**10C8**	9.40±1.20 ( n = 5 )	0/5	0	p = 0.043
**PBS**	7.67±0.75 ( n = 6 )	0/6	0	N/A^c^

a Guinea pigs were treated i.p. with 5 mg of the MAb as shown in the table on day 1 after infection with 1000 LD_50_ of the GA-ZEBOV. Survival of the guinea pigs was followed. The Student's T-test compared the MAb treatment group to the PBS control.

b Data for animals that died (number of animals in calculation).

c N/A: not applicable.

**Figure 2:**
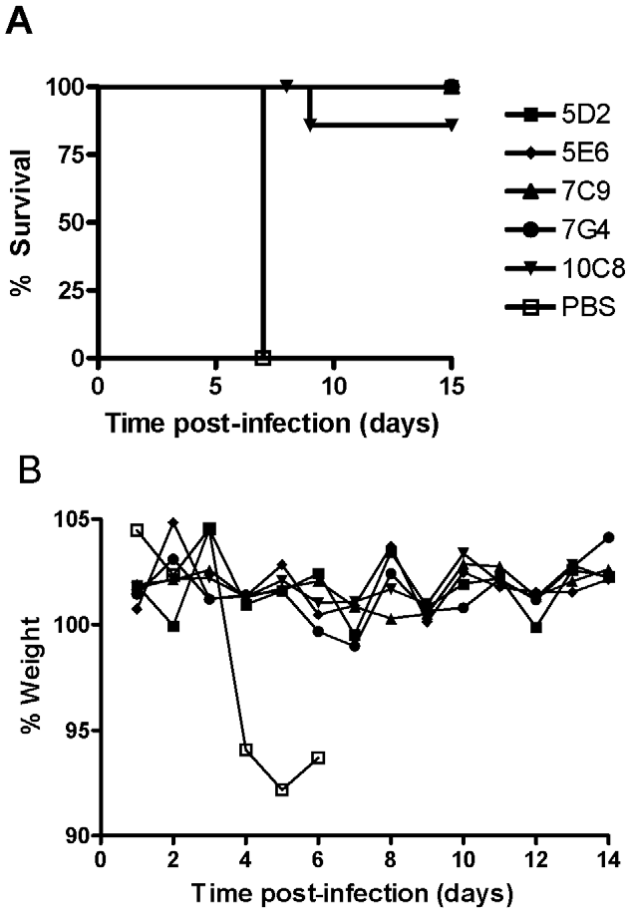
A treatment regimen using a combination of MAbs improves survival in GA-ZEBOV-infected guinea pigs. Guinea pigs were infected with 1000 LD_50_ of GA-ZEBOV i.p., then treated on 1 dpi with 3 mg of one of the non-neutralizing MAb i.p. (5D2, 5E6, 7C9, 7G4, or 10C8), followed by an i.p. treatment on 2 dpi with a combination of the 3 neutralizing MAbs ( 2 mg 4G7+1 mg 1H3+1 mg 2G4). The control animals received PBS i.p.. The percent survival (**A**) and average percent of the group weight (**B**) was determined.

Since individual MAbs were partially protective in the guinea pig, a second injection of the 3 neutralizing MAbs (1H3, 2G4, and 4G7) was included on 2 dpi (Figure 2). The guinea pigs were divided into 6 groups (n = 6), with one control group receiving only PBS, and 5 groups each receiving one of the non-neutralizing MAbs at 1 dpi, followed by the neutralizing MAb combination at 2 dpi. The PBS control treated animals all died with a mean time to death of 7 days. In contrast, all of the MAb treated groups demonstrated complete survival, except for 10C8 (83%). The treatment also improved morbidity as the MAb-treated groups maintained their weight in contrast to the controls that lost 6–7% of their weight by 4 and 5 dpi. This demonstrates that a combination of MAbs is an effective post-exposure treatment in guinea pigs.

### Neutralizing Mabs Alone Improve Survival in Guinea Pigs Infected with GA-ZEBOV

As two of the neutralizing antibodies were shown to be more effective at improving survival in guinea pigs (Table 4), the 3 ZEBOV GP-specific neutralizing MAbs were delivered as a combination alone to see if they would be sufficient as a therapy for an EBOV infection (Table 5). The combination of neutralizing MAbs (1.5 mg 1H3+1.5 mg 2G4+2 mg 4G7) was given to guinea pigs either 1 day before, or 1, 2 or 3 days after a 1000 LD_50_ GA-ZEBOV infection, and survival followed. The PBS control group all died, with a mean time to death of 6.58±0.59 days. In contrast all animals receiving the neutralizing MAb combination at 2 dpi. survived. When the treatment was given on 3 dpi the percent survival dropped to 66.7% with a mean time to death of 11.17±3.09 days. Receiving the combination either one day before or after resulted in a survival rate of 50%, with the meantime to death of 11.17±3.09 and 7.92±0.42, respectively. Overall, the neutralizing MAb combination improved survival in all treatment protocols with the 2 dpi treatment protocol being the most effective.

**Table 5:** Protective Efficacy of MAbs in Guinea Pigs Infected with GA-EBOV.

Treatment	Treatment time^a^	Meantime to death^b^ (days)	No. of survivors/total^c^	Survival (%)
**Neutralizing MAb Combination**	−1	11.17±3.09 (n = 3)	3/6	50
**Neutralizing MAb Combination**	+1	7.92±0.42 (n = 3)	3/6	50
**Neutralizing MAb Combination**	+2	N/A^d^	6/6	100
**Neutralizing MAb Combination**	+3	11.17±3.09 (n = 4)	4/6	67
**PBS**	+2	6.58±0.59 (n = 6)	0/6	0

a Guinea pigs were treated i.p. with a neutralizing MAb combination (2 mg 4G7+1.5 mg 1H3+1.5 mg 2G4) on the indicated days before or after challenge with 1000 LD_50_ GA-EBOV. The survival of the guinea pigs was followed.

b Average time to death. (No. of animals in calculation).

c Number of survivors at 28 dpi.

d N/A: not applicable.

## Discussion

In this study 8 ZEBOV GP-specific MAbs were tested for their efficacy in protecting against a ZEBOV infection in both a mouse and guinea pig model; and a post-exposure protocol for guinea pigs was optimized. Individually, each MAb extended survival partially, or completely after a lethal dose of MA-ZEBOV in mice, whereas only the 2G4 or 4G7 treated groups demonstrated a 60% survival rate against a GA-ZEBOV infection in guinea pigs. The dose response in the mouse experiment suggests some MAbs were more potent than others at improving survival, with 100% protection with 12.5 µg 5D2 in comparison to an 83% survival rate with 100 µg of 4G7 or 10C8 (Table 2). In general, the MAbs worked best for both animal models when given after the start of the infection, particularly at 1 and 2 dpi, before efficacy started to decrease at 3 dpi This suggests that there may be a limited time period in which to begin treatment after becoming infected. Within this 2 day time span, some MAbs were more effective when given at 1 dpi (5D2, 5E6, 7G4 and 10C8), and others at 2 dpi (1H3, 2G4, 4G7, and 7C9) in the mouse model. It is possible that since the MAbs and virus were both injected ip that the MAbs might inhibit ZEBOV infection of cells and extend life. As the MAbs were only partially effective when given individually in the guinea pig model, a combination of 3 neutralizing MAb (1H3, 2G4, 4G7) at 2 dpi was tested in guinea pigs and found to provide complete protection, and prevent morbidity. Each of these MAbs binds to different regions of GP1,2. 1H3 and 4G7 bind to the N- and C-terminus of GP1, respectively, whereas 2G4 binds to GP2 [36]. Targeting multiple regions of GP appears to be a successful strategy. It is possible that a variety of mechanisms for preventing infection are employed by the 8 MAbs that is reflected in the differing amounts of MAbs needed, and the time of treatment in which they are most effective. Some MAbs may have more affinity for their epitope, or the epitope may be more accessible in the natural conformation. There is precedence for this as two ZEBOV-specific neutralizing MAbs KZ52 and JP3K11 were found to neutralize ZEBOV by distinct mechanisms [42]. Based on the various studies using ZEBOV-specific MAbs as a therapy for EBOV infections, it appears that there is no way of predicting which MAb would provide complete protection. However, initial testing must begin in mice and guinea pigs in order to make the initial determination about what therapeutic approach might be best to test in NHPs and humans.

There have been several attempts at producing MAbs against ZEBOV GP however no clear pattern has emerged suggesting which primary sequence domain of GP is most immunogenic or whether neutralizing antibodies are more successful [26], [27], [31]–[34]. To date only neutralizing MAbs 133/3.16, 226/8.1 and KZ52 have shown the capacity to improve survival in guinea pigs [32], [33], [35]. MAbs 133/3.16 and 226/8.1 only provided partial protection, while 25 mg/kg of KZ52 was completely protective in guinea pigs when given at −1 or +1 hours, but had later failed to protect NHPs [26]. Individually, none of the MAbs in this study were as effective as KZ52 in the guinea pig model. However, a combination of MAbs was more effective, and the treatment could be delivered as late as 2 days after infection. This is a significant extension from the 1 hour post-infection required for KZ52. This is an important consideration as it is often difficult to begin treatment as early as 1 hour after an infection.

All 8 MAbs in this study were originally selected by screening for their SSS coating antigen [36]. Theoretically, the ability to bind to the natural conformation may be more advantageous as it is more likely that the epitopes would be available and not hidden, or that the MAbs would be able to interfere with events required for viral entry such as receptor binding, and membrane fusion. Preventing entry into the cell would decrease the overall infection levels thereby giving the immune system a better chance at controlling the infection. There are many characteristics that make an antibody effective and it may not be the same mechanism for any two MAbs. Overall, the MAbs generated in this study and the optimized protocols demonstrate their potential as a post-exposure therapeutic against a ZEBOV infection. Because previous KZ52 antibody treatments that proved effective in guinea pigs later failed to protect NHPs, it is vital that further evaluation of the neutralizing MAb combination protocol should be conducted in NHPs.
